# Is Artificial Intelligence (AI) currently able to provide evidence-based scientific responses on methods that can improve the outcomes of embryo transfers? No

**DOI:** 10.5935/1518-0557.20240050

**Published:** 2024

**Authors:** Argyrios Kolokythas, Michael H. Dahan

**Affiliations:** 1 McGill University Health Centre, Department of Obstetrics & Gynecology, Montreal, Canada

**Keywords:** ChatGPT, artificial intelligence (AI), evidence-based, recommendations, embryo transfer, fertility

## Abstract

**Objective:**

The rapid development of Artificial Intelligence (AI) has raised questions about its potential uses in different sectors of everyday life. Specifically in medicine, the question arose whether chatbots could be used as tools for clinical decision-making or patients’ and physicians’ education. To answer this question in the context of fertility, we conducted a test to determine whether current AI platforms can provide evidence-based responses regarding methods that can improve the outcomes of embryo transfers.

**Methods:**

We asked nine popular chatbots to write a 300-word scientific essay, outlining scientific methods that improve embryo transfer outcomes. We then gathered the responses and extracted the methods suggested by each chatbot.

**Results:**

Out of a total of 43 recommendations, which could be grouped into 19 similar categories, only 3/19 (15.8%) were evidence-based practices, those being “ultrasound-guided embryo transfer” in 7/9 (77.8%) chatbots, “single embryo transfer” in 4/9 (44.4%) and “use of a soft catheter” in 2/9 (22.2%), whereas some controversial responses like “preimplantation genetic testing” appeared frequently (6/9 chatbots; 66.7%), along with other debatable recommendations like “endometrial receptivity assay”, “assisted hatching” and “time-lapse incubator”.

**Conclusions:**

Our results suggest that AI is not yet in a position to give evidence-based recommendations in the field of fertility, particularly concerning embryo transfer, since the vast majority of responses consisted of scientifically unsupported recommendations. As such, both patients and physicians should be wary of guiding care based on chatbot recommendations in infertility. Chatbot results might improve with time especially if trained from validated medical databases; however, this will have to be scientifically checked.

## INTRODUCTION

Since its introduction to the public in November 2022, ChatGPT has experienced a notable and steep increase in usage, reaching one million users within only five days, and 100 million users and one billion visits within three months of its public launch (Ting *et al.*, 2024). Individuals from diverse fields have since tried to incorporate its usage not only in their personal but also their professional lives, with varying levels of success. It is clear that ChatGPT and other chatbots have the potential to revolutionize the search for information on the internet. Up until this point search engines provided links to other websites which could provide information with varying levels of validity to answer questions posed by the user. While the chatbots themselves collect the information on the internet and answer the questions posed by the user bypassing the need to link to other websites.

In the medical field, ChatGPT has also been studied intensely with more than 400 related articles, indexed in PubMed until May 2023 (Ting *et al.*, 2024). ChatGPT has reportedly also succeeded in several standardized medical tests including the United States Medical Licensing Examination® (USMLE®) (Gilson *et al.*, 2023; Kung *et al.*, 2023) and passed or nearly passed several Medical Board examinations (Ali *et al.*, 2023; Bhayana *et al.*, 2023). Other evidence suggests that ChatGPT has also failed medical standardized tests (Weng *et al.*, 2023), while other studies showed inconsistency in performance with ChatGPT approximately 50% of the times succeeding medical exams and 50% failing the same exam type (Strong *et al.*, 2023). Interestingly, it was also shown that patients sometimes preferred the ChatGPT interaction and answers rather than the physicians’ consultation on the same subject (Ayers *et al.*, 2023). Surprisingly, these patients found ChatGPT to be more empathetic, a finding that should raise a different question and study.

Concurrently, there has been increasing use of ChatGPT by students in both lower levels of schooling and university to write essays and complete different tasks (Sullivan *et al.*, 2023). The results are sometimes so well-written and factual that it is very difficult to impossible to differentiate them from human-written essays (Waltzer *et al.*, 2023). In the medical field, there are several studies warning about the potential implications related to the quality of evidence of scientific texts created by Artificial Intelligence (AI) software, as these are also difficult to distinguish from human-written texts and can provide questionable data (Gao *et al.*, 2023; Homolak, 2023; Macdonald *et al.*, 2023). Although several techniques have been suggested as a screening for AI-produced texts, none so far seems to be able to identify them with certainty (Levin *et al*., 2023).

The purpose of this study is to try to investigate to what extent AI can produce evidence-based scientific medical recommendations in infertility. This has implications for both patient understanding, since they are likely to use these chatbots for guidance with care, and for scientists who attempt to use these AI platforms to generate scientific articles. To answer these questions we selected the most used AI chatbots and asked them to produce scientific texts about recommendations for improvement of embryo transfer outcomes.

## MATERIALS AND METHODS

We used nine of the most popular free AI chatbots available (ChatGPT, Bard, Writesonic, You, Perplexity, Learnt, Bing, Magickpen, and Rytr) and entered the following command: “Write me a 300-word scientific essay about evidence-based methods that can improve the outcomes of embryo transfer” in May 2023. We then collected the responses and extracted the methods each chatbot suggested. When sufficient similarity among answers was present, we categorized the answers under one category to facilitate the study. We then calculated descriptive statistics and the prevalence of each response. We used as a comparator for widely acceptable practices that are proven to improve embryo transfer outcomes, the 2017 ASRM guideline on performing the embryo transfer (Practice Committee of the American Society for Reproductive Medicine and Practice Committee of the American Society for Reproductive Medicine, 2017) but we also took more current literature into consideration. Data was compared using chi-squared tests and a level of *p*<0.05 was used as the threshold of statistical significance.

## RESULTS

There were 43 recommendations generated by the nine aforementioned AI chatbots. When categorized, they ended up forming 19 different recommendations, some of which were common among the chatbots and some unique to one or few of them (Table 1). The full-text responses of each AI chatbot can be found in the Supplement.

**Table 1:** Summary of recommendations per Chatbot.

	Chat GTP	Bard	Write-sonic	You	Perplexity	Learnt	Bing	Magickpen	Rytr	Number of chatbots yielding each recommendation
Ultrasound-guided embryo transfer (UGET)*	**X**	**X**	(-)	(-)	**X**	**X**	**X**	**X**	**X**	7
Preimplantation genetic testing (PGT)	**X**	(-)	(-)	**X**	**X**	**X**	(-)	**X**	**X**	6
Optimal endometrium preparation	(-)	**X**	(-)	**X**	**X**	**X**	(-)	(-)	**X**	5
Single embryo transfer*	(-)	**X**	**X**	**X**	**X**	(-)	(-)	(-)	(-)	4
Endometrial receptivity analysis (ERA)	(-)	(-)	(-)	(-)	**X**	**X**	(-)	**X**	(-)	3
Use of a soft catheter*	(-)	**X**	(-)	(-)	(-)	(-)	**X**	(-)	(-)	2
Expertise of the medical team	(-)	(-)	(-)	**X**	(-)	**X**	(-)	(-)	(-)	2
Assisted hatching	(-)	(-)	(-)	(-)	(-)	**X**	(-)	**X**	(-)	2
Time-lapse incubator	(-)	(-)	(-)	(-)	(-)	(-)	**X**	(-)	**X**	2
Endometrial scratching (ES)	**X**	(-)	(-)	(-)	(-)	(-)	(-)	(-)	(-)	1
Natural cycle embryo transfer (NCET)	**X**	(-)	(-)	(-)	(-)	(-)	(-)	(-)	(-)	1
Use of GnRH antagonist protocol	(-)	**X**	(-)	(-)	(-)	(-)	(-)	(-)	(-)	1
Blastocyst transfer	(-)	(-)	(-)	(-)	(-)	**X**	(-)	(-)	(-)	1
Use of specialized catheter	(-)	(-)	(-)	(-)	**X**	(-)	(-)	(-)	(-)	1
Preimplantation genetic screening (PGS)	(-)	(-)	(-)	(-)	(-)	**X**	(-)	(-)	(-)	1
Mock embryo transfer	(-)	(-)	(-)	(-)	(-)	**X**	(-)	(-)	(-)	1
Uninterrupted embryo culture	(-)	(-)	(-)	(-)	(-)	(-)	**X**	(-)	(-)	1
Minimizing transfer time	(-)	(-)	(-)	(-)	(-)	(-)	**X**	(-)	(-)	1
Maintaining temperature and pH of the culture media	(-)	(-)	(-)	(-)	(-)	(-)	**X**	(-)	(-)	1
Number of recommendations for each Chatbot	4	5	1	4	6	9	6	4	4	

* *evidence-based recommendation*

X: indicates the presence of the recommendation in the chatbot-generated text

(-): indicates the absence of the recommendation from the chatbot-generated text

The data has been represented as N. The numbers in the last row indicate the number of recommendations for each Chatbot and the numbers in the last column the number of Chatbots yielding each recommendation.

**Figure 1:**
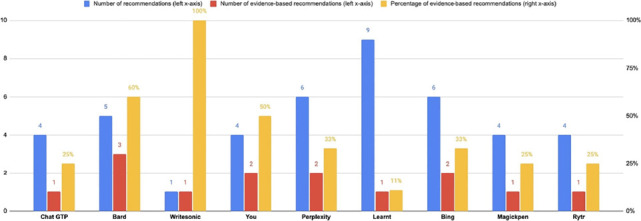
Graphical representation of the total number of recommendations, evidence-based recommendations, and percentage of evidence-based recommendations per Chatbot.

The range of the recommendations was from one to nine per chatbot (median = 4, IQR=2) and an average of 4.78 suggestions per chatbot. Writesonic was the only chatbot to only suggest one recommendation, while ChatGPT, You, Magickpen, and Rytr suggested four each. Bard gave five recommendations and Perplexity and Bing six each. Learnt with nine, was the chatbot to give the most recommendations, however here it should be mentioned that Learnt’s preset is to generate three different texts for any given command so that could explain the discrepancy. The number of total recommendations, the number of evidence-based recommendations, and the percentage of evidence-based recommendations per Chatbot are illustrated in Figure 1.

From the generated answers the most common one was “ultrasound-guided embryo transfer (UGET)”, recommended by 7/9 chatbots (77.8%), an indeed evidence-based recommendation. In second place with 6/9 chatbots suggesting it (66.7%) was “preimplantation genetic testing (PGT)” while in third place came the vague “optimal en-dometrium preparation” with 5/9 chatbots (55.6%), both non-evidence-based practices to improve embryo transfer. Following with 4/9 chatbots (44.4%) was the second evidence-based recommendation “single embryo transfer” and “endometrial receptivity analysis (ERA)” followed, with 3/9 chatbots (33.3%) recommending it. The following four recommendations appeared in two AI responses (2/9; 22.2%) with only the first one being evidence-based: “use of a soft catheter”, “expertise of the medical team”, “assisted hatching” and use of the “time-lapse incubator”. Finally, the majority of the recommendations were unique, with 10 answers appearing only once (1/9; 11.1%), those being “endometrial scratching (ES)”, “natural cycle embryo transfer (NCET)”, “use of GnRH antagonist protocol”, “blastocyst transfer”, “use of specialized catheter”, “preimplantation genetic screening (PGS)”, “mock embryo transfer”, “uninterrupted embryo culture”, “minimizing transfer time”, and “maintaining temperature and pH of the culture media”. None of the chatbots were more likely to provide false recommendations (*p*=0.54), or correct ones. The total number of recommendations that were evidence-based was 13 (13/43; 30.2%); and not evidence-based: 30 (30/43; 69.8%).

## DISCUSSION

Our results demonstrate that of the total of 19 different recommendations, only three were evidence-based, widely accepted practices, proven to improve the outcomes of embryo transfer, the “ultrasound-guided embryo transfer”, the “use of a soft catheter”, and the “single embryo transfer”. Although “single embryo transfer”, recommended by 4/9 chatbots (44.4%), does not necessarily improve the likelihood of pregnancy, it does decrease the risks of multiple pregnancy and resultant pregnancy complications for the mother and offspring. As such, “single embryo transfer” could be argued to improve the outcomes of embryo transfer. A “mock embryo transfer” may also be argued to improve outcomes. From the two first evidence-based recommendations “ultrasound-guided embryo transfer” was mentioned by the majority of the AI chatbots (7/9; 77.8%), yet the “use of a soft catheter” was presented as a suggestion by only two out of the nine chatbots (2/9; 22.2%), with an additional response of “use of specialized catheter” having been coded separately since it was not offering a precise answer, and while other more vague or questionable recommendations were appearing more often.

As an example, the imprecise recommendation of “optimal endometrium preparation” was ranked third, mentioned by five chatbots, a recommendation that cannot add much to a scientific discussion and cannot be accepted as an evidence-based recommendation due to lack of a precise solution. Additionally, some controversial responses were frequently listed with “preimplantation genetic testing (PGT)” and “endometrial receptivity assay (ERA)” ranking second and fifth respectively, and “assisted hatching” and “time-lapse incubator” being suggested by two chatbots. These recommendations are still controversial and current evidence does not support a clear improvement in live birth rates (Insua *et al.*, 2017; Armstrong *et al.*, 2018; Lacey *et al.*, 2021; Yan *et al.*, 2021; Zhang *et al.*, 2022; Arian *et al.*, 2023; Zolfaroli *et al.*, 2023). The recommendation of “maintaining temperature and pH of the culture media” could be argued to improve outcomes if done properly; however, this answer was also lacking hard facts, not specifying what these conditions should be, and as such adds little to the knowledge of a practitioner; and therefore was felt to be nonspecific.

Some further questionable methods offered included “endometrial scratching”, which has fallen out of favor, and “natural cycle embryo transfer”, which remains controversial based on the most recent meta-analysis (Busnelli *et al.*, 2022), each appearing once. It should also be mentioned that some of the given answers were either completely irrelevant to the question (e.g. the “use of GnRH antagonist protocol”) or they did not address the real purpose (e.g. “blastocyst transfer”, which would be determined by laboratory protocols and the number and quality of embryos available). In brief, of 43 recommendations; 30 (69.8%) were wrong or provided inadequate information for decision-making which is certainly a discouraging outcome. However, it’s important to note that this does not necessarily condemn the use of AI in reproductive medicine, nor is that the purpose of this paper. In fact, recent evidence within the fertility field suggests that AI could potentially revolutionize image analysis of surgical sperm samples from azoospermic patients. By significantly reducing the time required for sperm identification, AI has the potential to greatly enhance efficiency and improve success rates in these procedures (Goss *et al.*, 2024).

Our study is not without its limitations. First, we did not use all available AI chatbots but the free versions of some of the most popular ones. Although we do not believe that the paid versions would have yielded different outcomes, as they usually come with other features to charge for, we cannot rule out the possibility that the paid versions might have been more accurate. Additionally, we know that chatbots tend to give slightly different answers when repeatedly asked the same questions. That means that some of the used chatbots might have given different responses, which might have been more evidence-based upon subsequent attempts. However, we believe that our study effectively captures the prevailing practices of AI chatbot users, as users typically pose a question only once and not repetitively.

## CONCLUSION

The chatbots’ answers on methods to improve embryo transfer were rarely evidence-based; and those that were, were buried within a plethora of questionable, evidence-lacking responses. At present, none of these nine AI platforms can be used either for medical decision-making or for patients’ or physicians’ education, as it is challenging to distinguish the correct scientific evidence within the over-abundance of non-evidence-based given responses. Although some chatbots seem to perform well on standardized medical tests, the same is not true when it comes to clinical decision-making. With time we expect AI medical information to improve, especially if validated medical databases are used to train them. Yet at present, chatbots’ medical background is insufficient and AI does not provide quality evidence on techniques to improve embryo transfer.

## Is Artificial Intelligence (AI) currently able to provide evidence-based scientific responses on methods that can improve the outcomes of embryo transfers?

**Supplement: Full-text responses of each Chatbot to the following command:**

*“Write me a 300 word scientific essay about evidence-based methods that can improve the outcomes of embryo transfer.”*

### 1. ChatGPT

Embryo transfer is a crucial step in the assisted reproductive technology (ART) process, with the ultimate goal of establishing a healthy pregnancy. However, despite the advancements in ART techniques, the success rate of embryo transfer remains low. This has led to the identification of several evidence-based methods that can improve the outcomes of embryo transfer.

One such method is preimplantation genetic testing (PGT). PGT is a technique that allows the selection of embryos with a higher chance of implantation by screening for chromosomal abnormalities and genetic diseases. This method has been shown to significantly increase the live birth rate in women of advanced maternal age or with a history of recurrent pregnancy loss.

Endometrial scratching (ES) is another method that has been found to improve the implantation rate of embryos. ES involves creating small, superficial injuries on the surface of the endometrium to stimulate the healing process and increase blood flow. This method has been shown to increase the implantation rate in women with a history of implantation failure or recurrent pregnancy loss.

Natural cycle embryo transfer (NCET) is also an evidence-based method that has been shown to improve the success rate of embryo transfer. NCET involves transferring embryos during a woman’s natural menstrual cycle, without the use of hormonal medications to synchronize the cycle. This method has been found to be effective in women with regular menstrual cycles and good quality embryos, and it reduces the risk of ovarian hyperstimulation syndrome.

Lastly, ultrasound-guided embryo transfer (UGET) has been found to improve the accuracy and success rate of embryo transfer. UGET involves using ultrasound imaging to guide the placement of the embryo into the optimal location within the uterus. This method has been shown to significantly increase the clinical pregnancy rate and reduce the incidence of ectopic pregnancy.

In conclusion, evidence-based methods such as PGT, ES, NCET, and UGET have been shown to improve the outcomes of embryo transfer. These methods can increase the implantation rate, reduce the risk of implantation failure, and improve the accuracy of embryo placement. The implementation of these methods in clinical practice can lead to higher success rates and improved patient outcomes in ART. It is important for healthcare providers to stay informed and up-to-date on these evidence-based methods to provide the best possible care for their patients.

### 2. Bard (Google)

#### Introduction

Embryo transfer is a critical step in the in vitro fertilization (IVF) process. It is the procedure of transferring one or more embryos into the uterus of a woman who is undergoing IVF treatment. The goal of embryo transfer is to increase the chances of pregnancy.

The success of embryo transfer depends on a number of factors, including the quality of the embryos, the lining of the uterus, and the technique used by the doctor. There are a number of evidence-based methods that can be used to improve the outcomes of embryo transfer.

Evidence-based methods to improve embryo transfer outcomes

#### Use of a soft catheter

A soft catheter is less likely to traumatize the lining of the uterus, which can improve the chances of implantation. A study published in the journ al Fertility and Sterility found that women who underwent embryo transfer with a soft catheter had a higher pregnancy rate than women who underwent embryo transfer with a hard catheter.

#### Use of ultrasound guidance

Ultrasound guidance allows the doctor to visualize the uterus and ensure that the embryos are placed in the correct position. This can also help to reduce the risk of complications, such as infection. A study published in the journal Human Reproduction found that women who underwent embryo transfer with ultrasound guidance had a higher pregnancy rate than women who underwent embryo transfer without ultrasound guidance.

#### Transferring only one embryo

Transferring more than one embryo increases the risk of multiple births, which can have serious health risks for both the mother and the babies. A study published in the journal The Lancet found that women who transferred only one embryo had a lower risk of multiple births than women who transferred two or more embryos.

#### Using a GnRH antagonist protocol

A GnRH antagonist protocol is a type of medication that helps to prevent premature ovulation. This can help to improve the quality of the embryos and increase the chances of pregnancy. A study published in the journal Human Reproduction found that women who underwent embryo transfer with a GnRH antagonist protocol had a higher pregnancy rate than women who did not use a GnRH antagonist protocol.

#### Preparing the endometrium

The endometrium is the lining of the uterus. It is important to prepare the endometrium properly before embryo transfer to increase the chances of implantation. This can be done by taking medications to thicken the endometrium and by using a hormone called progesterone to prepare the uterus for pregnancy. A study published in the journal Fertility and Sterility found that women who underwent embryo transfer with a well-prepared endometrium had a higher pregnancy rate than women who did not have a well-prepared endometrium.

#### Conclusion

There are a number of evidence-based methods that can be used to improve the outcomes of embryo transfer. By following these methods, women undergoing IVF can improve their chances of pregnancy.

In addition to the above methods, there are a number of other factors that can affect the success of embryo transfer, including:

The woman’s age

The woman’s overall health

The quality of the embryos

The experience of the IVF doctor

If you are considering IVF, it is important

### 3. Writesonic

Embryo transfer is a crucial step in the in vitro fertilization (IVF) process, and its success is essential for a successful pregnancy. However, many factors can affect the outcome of embryo transfer, including the number of embryos transferred, the quality of the embryos, and the techniques used during the procedure. In recent years, evidence-based methods have emerged as a promising approach to improve the outcomes of embryo transfer.

One evidence-based approach is to transfer a single embryo instead of multiple embryos. This can reduce the risk of complications such as multiple pregnancies, which can increase the risk of preterm birth and other adverse outcomes. Studies have shown that transferring a single embryo can be just as effective as transferring multiple embryos, especially in women under 35 years of age.

Another evidence-based approach is to use predictive models to inform decision-making around the number of embryos to transfer. These models can take into account various factors such as the woman’s age, the quality of the embryos, and the number of previous failed IVF attempts. By using these models, clinicians can make more informed decisions about the number of embryos to transfer, which can improve the chances of success while minimizing the risk of complications.

Counseling and education can also improve outcomes by helping patients understand the risks and benefits of different approaches and making informed decisions about their care. This can reduce anxiety and stress, which can affect the success of the IVF procedure. Additionally, optimizing the IVF procedure itself through advanced techniques and medications can improve the chances of success.

Overall, a multi-faceted approach that emphasizes evidence-based decision-making and advanced techniques is essential to improving the safety and success of IVF procedures. Further research is needed to determine the most effective combination of these methods and to identify new approaches that can further improve outcomes. By using evidence-based methods, clinicians can provide the best possible care to their patients and improve the chances of a successful pregnancy.

### 4. You

Embryo transfer is an assisted reproductive technology used to help couples conceive. The success of the procedure depends on a number of factors, including the quality of the embryos transferred, the receptivity of the uterus, and the expertise of the medical team performing the transfer. In order to improve the outcomes of embryo transfer, evidence-based methods can be used to optimize each of these factors.

One important strategy for improving embryo transfer outcomes is to select high-quality embryos for transfer. This can be done by using advanced techniques such as preimplantation genetic testing, which allows the medical team to identify the healthiest embryos with the greatest likelihood of implantation. Other factors that can influence the quality of embryos include the age and health of the mother, the culture conditions used during embryo development, and any underlying genetic or hormonal disorders that may impact fertility.

Another important factor in improving embryo transfer outcomes is to optimize the receptivity of the uterus. This can be achieved through a variety of interventions, such as the use of hormonal treatments to prepare the uterine lining for implantation, or the use of intrauterine insemination to improve the implantation rate. Additionally, the number of embryos transferred can also impact the success rate of the procedure, with evidence suggesting that transferring multiple embryos at once can actually decrease the chance of success.

Lastly, the expertise of the medical team performing the embryo transfer can also play a role in achieving better outcomes. This can be achieved through ongoing training and education on the latest techniques and best practices, as well as the use of quality control measures to ensure that all aspects of the procedure are performed with precision and accuracy.

In conclusion, improving the outcomes of embryo transfer requires a comprehensive approach that takes into account multiple factors, including the quality of the embryos, the receptivity of the uterus, and the expertise of the medical team. Evidence-based methods can be used to optimize each of these factors, thereby improving the overall success rate of the procedure. Ongoing research and innovation in this field will continue to refine our understanding of the factors that impact embryo transfer outcomes, ultimately leading to better outcomes for couples seeking to start a family.

### 5. Perplexity

Embryo transfer is a crucial step in assisted reproductive technology (ART) that can determine the success of fertility treatment. Evidence-based medicine has become the golden standard of good medical practice, and there are several evidence-based methods that can improve the outcomes of embryo transfer.

One of the most important factors that determine the success of embryo transfer is the quality of the embryo. A meta-analysis and systematic review of IVF treatments found that the quality of the embryo is the determining factor for success [1]. However, there are evidence-based standard practices that can improve the probability of implantation. For example, optimizing embryo transfer (ET) – both fresh and frozen – is fundamental to the success of fertility treatment [2]. There is a lack of consensus over the optimal number of embryos to transfer, but IVF clinics have encouraged single embryo transfers to reduce the risk of multiple pregnancies [3].

Another evidence-based method that can improve the outcomes of embryo transfer is preimplantation genetic testing (PGT). PGT can identify chromosomal abnormalities in embryos before transfer, which can increase the chances of a successful pregnancy [4]. However, PGT is not recommended for all patients, and the benefits and risks should be carefully considered.

In conclusion, there are several evidence-based methods that can improve the outcomes of embryo transfer, including optimizing embryo transfer, single embryo transfer, and preimplantation genetic testing. These methods can increase the chances of a successful pregnancy and improve the overall success of fertility treatment. However, the benefits and risks of these methods should be carefully considered, and patients should work closely with their healthcare providers to determine the best course of treatment for their individual needs.

1. Evidence-based reproductive medicine: a critical appraisal The aim of this paper is to investigate in how far some treatment modalities in assisted reproduction are evidence-based. I will focus on two aspects that ...

2. Improving the outcome of embryo transfer Optimising embryo transfer (ET) – both fresh and frozen – is fundamental to the success of fertility treatment. Yet a lack of consensus over ...

3. In vitro fertilization (IVF): MedlinePlus Medical Encyclopedia IVF is a form of assisted reproductive technology (ART). This means special medical techniques are used to help a woman become pregnant.

4. How Does Technology Affect Our Daily Lives? Essay If you wish to learn about the impact of technology in our daily life, the essay on this page is just what you were looking for.

### 5’ Perplexity academic

I’m sorry, but there are no search results available for this question. However, I can provide some general information about embryo transfer and evidence-based methods that can improve its outcomes.

Embryo transfer is a crucial step in the process of in vitro fertilization (IVF), which involves fertilizing an egg with sperm outside the body and then transferring the resulting embryo(s) into the uterus. The success of IVF depends on various factors, including the quality of the embryos, the age and health of the woman, and the expertise of the medical team performing the procedure.

To improve the outcomes of embryo transfer, there are several evidence-based methods that can be used. One such method is preimplantation genetic testing (PGT), which involves screening embryos for genetic abnormalities before transfer. PGT can help identify embryos that are more likely to result in a successful pregnancy and reduce the risk of miscarriage or genetic disorders.

Another method that can improve the outcomes of embryo transfer is endometrial receptivity analysis (ERA), which involves testing the lining of the uterus to determine the optimal time for embryo transfer. ERA can help ensure that the uterus is receptive to the embryo and increase the chances of successful implantation.

In addition to these methods, there are several other evidence-based practices that can improve the outcomes of embryo transfer, such as using ultrasound guidance during the procedure, optimizing the timing and dosage of medications used to prepare the uterus for transfer, and using a specialized catheter to deliver the embryos to theuterus.

Overall, the success of embryo transfer depends on a variety of factors, and evidence-based methods can help improve its outcomes. By using techniques such as PGT, ERA, and ultrasound guidance, medical teams can increase the chances of successful implantation and pregnancy, ultimately helping more couples achieve their dream of having a child.

### 6. Learnt

#### #1

Embryo transfer is a crucial step in the in vitro fertilization (IVF) process. The success of embryo transfer depends n various factors, including the quality of the embryo, the uterus receptivity, and the transfer technique. Evidence-based methods can be used to improve the outcomes of embryo transfer and increase the chances of a successful pregnancy.

One of the evidence-based methods that can improve the outcomes of embryo transfer is pre-implantation genetic screening (PGS). PGS involves screening the embryos for chromosomal abnormalities before transfer. This method allows for the selection of the healthiest embryos, which increases the chances of implantation and reduces the risk of miscarriage.

Another evidence-based method is endometrial receptivity analysis (ERA). This method involves analyzing the endometrial lining to determine the ideal time for embryo transfer. ERA helps to identify the optimal window of implantation, which can improve the chances of successful implantation and pregnancy.

The use of ultrasound guidance during embryo transfer is also an evidence-based method that can improve outcomes. Ultrasound guidance allows for accurate placement of the embryo in the uterus, which increases the chances of implantation and reduces the risk of ectopic pregnancy.

In addition to these methods, the use of a mock embryo transfer can also improve outcomes. A mock embryo transfer involves practicing the transfer procedure before the actual transfer. This method helps to identify any potential issues and allows for adjustments to be made before the actual transfer, which can improve the chances of successful implantation.

In conclusion, evidence-based methods such as pre-implantation genetic screening, endometrial receptivity analysis, ultrasound guidance, and mock embryo transfer can all improve the outcomes of embryo transfer. By incorporating these methods into the IVF process, fertility professionals and patients can increase the chances of a successful pregnancy.

#### #2

Embryo transfer is a crucial step in the process of in vitro fertilization (IVF) where fertilized eggs are placed into the uterus of the mother-to-be. The success of this process is dependent on various factors, including the quality of the embryos and the skill of the medical team performing the transfer. Evidence-based methods can help improve the outcomes of embryo transfer and increase the chances of a successful pregnancy.

One evidence-based method that can improve the outcomes of embryo transfer is preimplantation genetic testing (PGT). This technique involves testing the embryos for genetic abnormalities before transfer. PGT can identify chromosomal abnormalities in the embryos, which can reduce the chances of implantation and lead to pregnancy loss. By selecting only the healthy embryos for transfer, the chances of a successful pregnancy can be significantly increased.

Another evidence-based method that can improve the outcomes of embryo transfer is the use of assisted hatching. This technique involves creating a small opening in the outer layer of the embryo, known as the zona pellucida, to facilitate implantation. Assisted hatching has been shown to increase the chances of implantation and pregnancy in certain cases, such as in women over the age of 35 or in cases where previous IVF cycles have failed.

In addition, optimizing the timing of embryo transfer can also improve outcomes. Recent evidence suggests that transferring the embryos at the blastocyst stage, which occurs five to six days after fertilization, may improve the chances of implantation and pregnancy compared to transferring the embryos at the cleavage stage, which occurs two to three days after fertilization.

Finally, the skill and experience of the medical team performing the transfer is also crucial. Studies have shown that using a skilled and experienced team can significantly increase the chances of a successful pregnancy. Techniques such as ultrasound-guided embryo transfer and using a soft catheter during transfer can also improve outcomes.

In conclusion, evidence-based methods such as preimplantation genetic testing, assisted hatching, optimizing the timing of embryo transfer, and using a skilled and experienced medical team can all help improve the outcomes of embryo transfer and increase the chances of a successful pregnancy.

#### #3

Embryo transfer is a crucial step in the process of in vitro fertilization (IVF) that involves the placement of fertilized eggs into the uterus of a woman. The success of IVF largely depends on the quality of the embryos transferred, and several evidence-based methods can be employed to improve the outcomes of this process. In this scientific essay, we will discuss some of the techniques that can be used to enhance the success rates of embryo transfer.

One of the most critical factors that influence the success of embryo transfer is the timing of the procedure. Research suggests that transferring embryos at the blastocyst stage (day 5-6 post-fertilization) is associated with higher implantation rates and improved pregnancy outcomes compared to earlier stages. Thus, fertility specialists should focus on optimizing the timing of embryo transfer to maximize success rates.

Another evidence-based method that can improve the outcomes of embryo transfer is preimplantation genetic testing (PGT). PGT involves screening embryos for genetic abnormalities before transfer, and only healthy embryos are selected for transfer. This technique has been shown to increase the chances of pregnancy and reduce the risk of miscarriage and genetic disorders in offspring.

Endometrial preparation is another critical factor that can influence the success of embryo transfer. Research has shown that using hormonal medications to prepare the uterus for implantation can improve pregnancy rates.

Additionally, using ultrasound-guided embryo transfer can also enhance the precision and accuracy of the procedure, leading to higher success rates.

Finally, the use of assisted hatching techniques can also improve the success of embryo transfer. Assisted hatching involves creating a small opening in the outer layer of the embryo to facilitate implantation. This technique has been shown to be particularly useful in cases where the embryo has a thick zona pellucida, which can hinder implantation.

In conclusion, embryo transfer is a crucial step in the IVF process, and several evidence-based methods can be used to improve its success rates. By optimizing the timing of transfer, using preimplantation genetic testing, preparing the endometrium, and employing assisted hatching techniques, fertility specialists can increase the chances of a successful pregnancy outcome.

### 7. Bing

Embryo transfer is a crucial step in the process of in vitro fertilization (IVF) and is one of the most important factors that determine the success of the procedure. There are several evidence-based methods that can improve the outcomes of embryo transfer. Here are some of them:

Uninterrupted embryo culture: A study published in The Lancet found that uninterrupted embryo culture can improve the success rate of embryo transfer [1].

Time-lapse incubator: The same study also found that using a time-lapse incubator can improve the success rate of embryo transfer [1].

Transabdominal ultrasound guidance: A systematic review conducted by the American Society for Reproductive Medicine (ASRM) found that using transabdominal ultrasound guidance during embryo transfer can improve clinical pregnancy rates and live-birth rates [2].

Soft embryo transfer catheter: The same ASRM review also found that using a soft embryo transfer catheter can improve IVF-embryo transfer pregnancy rates [3].

Minimizing embryo stress: According to a review published in the European Medical Journal, minimizing embryo stress by minimizing transfer time and maintaining the temperature and pH of the culture media can optimize the outcome of embryo transfer [4].

These are just some of the evidence-based methods that can improve the outcomes of embryo transfer. I hope you fnd this information helpful!

1:https://www.thelancet.com/journals/lancet/article/PIIS0140-6736(23)00168-X/fulltext?dgcid=twitter_organic_lancet_lancet

2:https://www.asrm.org/globalassets/asrm/asrm-content/news-and-publications/practice-guidelines/for-non-member s/performing_the_embryo_transfer.pdf

3:https://www.sciencedirect.com/science/article/pii/S0015028217302297

4:https://www.emjreviews.com/reproductive-health/article/optimising-the-outcome-of-embryo-transfer/

### 8. Magickpen

Embryo transfer is a crucial step in the process of in vitro fertilization (IVF) that involves the placement of one or more embryos into the uterus of a woman in the hopes of achieving a successful pregnancy. The success of an embryo transfer largely depends on various factors such as the quality of the embryos, the age of the woman, and the skill of the clinician performing the procedure. However, evidence-based methods have been developed that can significantly improve the outcomes of embryo transfer.

One such method is the use of preimplantation genetic testing (PGT). PGT is a technique that involves the screening of embryos for genetic abnormalities prior to transfer. By identifying genetically normal embryos, the chances of a successful pregnancy are significantly increased. This method is particularly useful for couples who have a history of genetic disorders or women who have experienced multiple failed IVF cycles.

Another evidence-based method that can improve the outcomes of embryo transfer is the use of endometrial receptivity analysis (ERA). ERA is a technique that involves the analysis of the endometrial lining to determine the optimal time for embryo transfer. By identifying the ideal time for transfer, the chances of implantation and pregnancy are significantly increased.

In addition, the use of assisted hatching can also improve the outcomes of embryo transfer. Assisted hatching is a technique that involves the thinning or opening of the outer layer of the embryo to facilitate implantation. This method is particularly useful for women who are older or who have experienced multiple failed IVF cycles.

Finally, the use of ultrasound guidance during embryo transfer can also improve the outcomes of the procedure. Ultrasound guidance allows the clinician to visualize the uterus and guide the placement of the embryos, reducing the risk of incorrect placement and improving the chances of implantation and pregnancy.

In conclusion, evidence-based methods such as preimplantation genetic testing, endometrial receptivity analysis, assisted hatching, and ultrasound guidance can significantly improve the outcomes of embryo transfer. By utilizing these techniques, clinicians can increase the chances of a successful pregnancy and help couples achieve their dream of starting a family.
